# The Causation of Apoplexy

**Published:** 1914-08-08

**Authors:** 


### The Causation of Apoplexy.

If the views of Dr. W. B. Cadwaladar, as expounded in the *Journal of the American Medical Association*, find favour with neurologists, some of the teaching about hemiplegia which appears in every textbook will have to be revised. The three lesions which may produce this condition are, of course, intracerebral hæmorrhage, embolism, and thrombosis. The treatment when the last of these is diagnosed is almost the exact opposite of that recommended when the first occurs, and the books give a list of the criteria by which the two conditions may be distinguished. Yet Dr. Cadwaladar now declares that cerebral hæmorrhage is practically always fatal. When repeated attacks of apoplexy occur, the ultimately fatal attack may be due either to hæmorrhage or thrombosis; but the previous attacks are invariably caused by softening (from thrombosis) and not by hæmorrhage. Nor is there, according to this author, any distinction in type between the apoplexy of hæmorrhage and that of softening. The onset and character may be exactly alike, although the lesions and their methods of causation are so totally different. It is true that a sudden onset with rapidly developing and persistent coma usually means hæmorrhage, but so too may a slow onset with premonitory symptoms without profound coma. Ingravescent apoplexy, indeed, is more likely to be due to hæmorrhage than to thrombosis. It is obvious that if these views are correct the accepted treatment of apoplexy will have to be greatly modified.

